# Important steps for PrEP uptake among adolescent men who have sex with men and transgender women in Brazil

**DOI:** 10.1371/journal.pone.0281654

**Published:** 2023-04-04

**Authors:** Fabiane Soares, Laio Magno, Marcos Eustorgio Filho, Filipe Mateus Duarte, Alexandre Grangeiro, Dirceu Greco, Inês Dourado, PrEP1519 Brazil study group

**Affiliations:** 1 Institute of Collective Health, Federal University of Bahia, Salvador, Bahia, Brazil; 2 Department of Life Sciences, State University of Bahia, Salvador, Bahia, Brazil; 3 School of Preventive Medicine, University of São Paulo, São Paulo, São Paulo, Brazil; 4 School of Medicine, Federal University of Minas Gerais, Belo Horizonte, Minas Gerais, Brazil; University of California San Diego, UNITED STATES

## Abstract

HIV Pre-exposure prophylaxis (PrEP) is an effective prevention tool, but there are still few studies about PrEP uptake among adolescents. We aimed to analyze the PrEP uptake process and factors associated with daily oral PrEP initiation among adolescent men who have sex with men (aMSM) and transgender women (aTGW) in Brazil. Baseline data from the first demonstration PrEP cohort study among aMSM and aTGW 15–19 years old (yo) ongoing in three large Brazilian cities (PrEP1519). After completing informed consent procedures, participants were enrolled in the cohort from February/2019 to February/2021. A socio-behavioral questionnaire was applied. Factors associated with PrEP initiation were assessed using a logistic regression model with adjusted prevalence ratios (aPR) and 95% confidence intervals (95%CI). Among recruited participants, 174 (19,2%) were aged 15–17 yo and 734 (80,8%) 18–19 yo. The rate of PrEP initiation was 78.2% and 77.4% for 15–17 yo and 18–19 yo, respectively. Factors associated with PrEP initiation were: black or mixed race (aPR 2.31; 95%CI: 1.10–4.84) among the younger adolescents 15–17 yo; experienced violence and/or discrimination due to their sexual orientation or gender identity (aPR 1.21; 95%CI: 1.01–1.46); transactional sex (aPR 1.32; 95%CI: 1.04–1.68); and having had between 2 to 5 sexual partners in the previous three months (aPR 1.39; 95%CI: 1.15–1.68) among those 18–19 yo. Unprotected receptive anal intercourse in the previous six months was associated with PrEP initiation in both age groups (aPR 1.98; 95%CI: 1.02–3.85 and aPR 1.45; 95%CI: 1.19–1.76 among 15–17 yo and 18–19 yo, respectively). The biggest challenge to promoting PrEP use for aMSM and aTGW was in the first steps of the PrEP uptake process. Once they were linked to the PrEP clinic, initiation rates were high.

## Introduction

In Brazil, the HIV epidemic disproportionately affects gays and other men who have sex with men (MSM) and transgender women (TGW) [1–5]. Studies show that these populations face barriers to gaining access to HIV prevention and care services [5–7]. In Brazil, respondent-driven sampling (RDS) surveys reported a high prevalence of HIV among adults MSM [8, 9] and TGW [1, 10], approaching 18% and 30%, respectively. In recent years, an increase in HIV incidence among young and adolescent MSM and TGW have been observed [11–14]. In addition, recent data from the Brazilian Ministry of Health Epidemiological Bulletin, based on cases reported in health care services, show an increase of 64.9% and 74.8% in AIDS incidence rates among young men aged 15 to 19 years and 20 to 24 years, respectively, from 2009 to 2019. In 2019, the highest estimated rate was 52.0 cases per 100,000 among people aged 25 to 29 years, which exceeded the rates in men aged 30 to 39 years, which were higher until 2015. And an increased trend from 2000 onwards is observed among adolescents from key populations (KP), such as MSM and TGW [15].

Despite these alarming statistics, there has been a dearth of effective HIV prevention interventions for this population [16]. Among adolescent MSM (aMSM) and TGW (aTGW), many intersecting vulnerabilities expose them to greater HIV risk [14], amongst them discrimination due to their sexual orientation and gender identity, experiences of stigma and violence [17–19], socioeconomic and political power inequalities [19, 20], and low access to education, especially to sex education specific to sexual or gender minorities youth [16, 20, 21].

Daily oral pre-exposure prophylaxis (PrEP) is an effective component of combination HIV prevention and is based on the use of an oral fixed-dose of a combination of nucleoside analogs antiretroviral-tenofovir (TDF) and emtricitabine (FTC), co-formulated in a single pill. The World Health Organization (WHO) recommends PrEP for adults and adolescent MSM and TGW at increased risk for HIV infection as an additional prevention strategy [22, 23]. Its high efficacy has been proven in clinical trials upon adherence to daily pill use [24–26]. In demonstration trials, PrEP was shown effective and safe for HIV prevention among MSM and TGW adults and adolescents [27–29].

PrEP was introduced in the Brazilian National Health System (in Portuguese–Sistema Único de Saúde—SUS) in late 2017 for population groups at a higher risk of infection [30]. PrEP is also safe and effective for adolescents from key populations (KP) [31] and was approved for adolescents by the US Food and Drug Administration in 2018 [32]. Recently, in 2022, it was released in Brazil for these adolescents and other people who are at risk for HIV infection over 15 years old, weighing at least 35 kg (77 lb).

PrEP access and use are processes that involve different steps, from assessing the HIV risk of potential users, promoting information, accessing and connecting them to health services, to prescribing and beginning use (PrEP uptake process) [33, 34].

Currently, PrEP prescriptions are suggested on the same day as the clinical checkup takes place, with a negative rapid test for HIV, while waiting for the results of safety laboratory tests is not mandatory [30, 35]. This strategy is considered safe and adopted by PrEP programs in Latin American countries and the United States of America (USA), in addition to being effective for the expansion of PrEP uptake [36–40].

However, there is limited knowledge about PrEP uptake among adolescent MSM and TGW. Studies with this population are still scarce and have small sample sizes [41–43], especially among adolescents younger than 18 years old (yo). The decision to use PrEP is determined by factors related to the prophylaxis characteristics and a set of individual, family, interpersonal, contextual, and structural conditions [44]. Thus, different age groups of adolescents will have different experiences and considerations about PrEP, and specific factors may be associated with PrEP initiation according to age groups. This article aims to describe the PrEP uptake process and analyze predictors for PrEP initiation among adolescent MSM and TGW in Brazil.

## Material and methods

### Study design

For this analysis, we used data from the baseline of the PrEP1519 study, the first cohort to demonstrate the effectiveness of PrEP in Brazil among aMSM and aTGW 15–19 year old at high risk of HIV infection, ongoing in three major Brazilian capitals: in Salvador (located at a Diversity Center that advocates for the rights of Lesbian, Gay, Bisexual, Transgender, Queer, Intersexual, Asexual and others (LGBTQIA+), in Belo Horizonte (located at a youth reference center), and in São Paulo (located in an HIV testing and counseling center), the last two in the public health system. The location of the PrEP clinics was carefully chosen, considering the accessibility and demands of sexual minorities adolescents to facilitate their access and linking to the services.

The eligibility criteria for enrollment in the cohort were: self-identification as MSM or TGW; 15–19 years old at the time of the study admission; sexual practices with cisgender men, and/or TGW; reside, work or study in one of the study cities, and test HIV negative at the time of enrollment. MSM and TGW were excluded if they were under the effect/influence of drugs and alcohol during the interview or had a mental illness that made it difficult to understand the research questions and the need for daily PrEP use. Upon eligibility criteria and the proposed steps for the study were informed, those who agreed to participate signed an informed consent (according to the court orders decisions defined for each city), were tested at the initial visit (baseline) for HIV using a 4th generation Rapid Test (Ag/Ab) followed by another 3rd generation Rapid Test and serological tests for other sexually transmitted infections (STIs). Participants self-selected into one out of two arms to participate in the study: (a) the PrEP arm included those that enrolled in daily use of oral PrEP with the TDF/FTC combination; (b) the non-PrEP arm included those who were not eligible for PrEP, and those who were eligible but chose not to use the prophylaxis and opted to receive only other HIV combination prevention methods (counseling, condoms, lubricant, HIV post-exposure prophylaxis (PEP) and HIV self-test).

After enrollment in the cohort, the participants were followed by a multidisciplinary team composed of physicians, nurses, social workers, psychologists, and pharmacists on regular visits or visits scheduled by the adolescents´ demand. The participants are also assigned a study peer-navigator and are monitored by the health team. These activities take place in person, via smartphone, and over WhatsApp, Instagram, and Facebook messages.

### Theoretical framework

The PrEP uptake amongst adolescents in this study included necessary steps before PrEP initiation: (i) *Facilitating PrEP access–*strategies that facilitate access to PrEP for adolescents at higher risk of HIV; (ii) *Linking to PrEP care–*linking to the study PrEP clinics; *(iii) Intention to use PrEP–*willingness to use PrEP; *(iv) Eligible for PrEP–*meeting the clinical criteria for PrEP use; *(v) Initiating PrEP–*the adolescent possesses the PrEP pills and starts using them (adapted from Nunn [34]). Next, we describe the PrEP uptake process for participants enrolled in PrEP.

#### Facilitating PrEP access

The aMSM and aTGW were recruited by young peer educators (PE) through different strategies: mobilization activities in places where adolescents meet, such as high schools, universities, public squares, bars, parks and beaches; intervention on social media such as Instagram, Facebook, WhatsApp, Twitter, Youtube; as well as hook-up apps such as Grindr, Tinder, and Badoo. During recruitment, the PE guided on the importance of combined HIV prevention, PrEP, scheduled clinical checkups, and guidance on how to get to the service. In addition, adolescents could also spontaneously attend clinics or were referred by other health services, friends, sexual partners, and Non-Governmental Organizations (NGOs) LGBTQIA+ [45]. Recruited aMSM and aTGW who arrived at PrEP clinics and underwent clinical examination by members of the multidisciplinary team for HIV risk and vulnerability assessments, counseling on combination prevention and clinical follow-up. During scheduled visits, participants had available reimbursement of BRL 30.00 for transportation and food costs.

#### Linking to PrEP care

Upon enrolling in the project, the adolescents were assisted by a multidisciplinary team and were instructed about HIV prevention measures, including PrEP. The most appropriate prevention strategies for each adolescent were evaluated during clinical care, along with the participants, based on their vulnerability to STIs, clinical condition, and willingness to use them. At this time, participants could choose to enroll in the PrEP or non-PrEP arm.

#### Intention to use PrEP

Participants were asked about their willingness to use PrEP during clinical care after the provider explained the PrEP use, side effects, and effectiveness.

#### PrEP eligibility

For PrEP initiation, besides the intention to use, the participants had to meet clinical criteria, which includes at least one of the following: unprotected anal sex in the last six months, episode of STI and use of HIV PEP in the last 12 months, frequent use of alcohol or drugs before or during sexual intercourse (chemsex), reports of transactional sex (sex in exchange for money or favors), or any specific situation shared between the adolescent and interviewer, considered vulnerable to HIV and other STIs. Those who have renal impairment (defined by Glomerular Filtration Rate < 60 ml/min/1.75m2, using the Cockcroft-Galt formula for people over 17 years and Schwartz formula for people under 17 years), history of spontaneous bone fracture, clinical condition suggesting acute retroviral syndrome in the last 30 days or risky sexual intercourse in the last 72 hours, in the latter case, immediately referred for PEP use and were temporarily or permanently excluded from the group using PrEP. Laboratory and clinical criteria were evaluated after 30 days, with the availability of results.

#### Prescribing and initiating PrEP

Participants eligible for PrEP who chose to use it initiated PrEP on the same day of their first visit to the clinic. The PrEP prescription happens after the rapid test for HIV, with a non-reactive result, and the collection of biological material for tests to monitor the safety of drug use. During the clinical visit, health professionals advise participants on the importance of adherence and side effects, answer questions and concerns about using of PrEP. At the end of the visit, adolescents received a PrEP prescription and a bottle with 30 pills for one month. Follow-up visits were scheduled quarterly after the first visit (three months, six months, nine months, and so on).

### Data collection

This analysis used the cohort baseline data from February 2019 to February 2021 and three sources of information: a) participant registration form with sociodemographic information filled out upon admission to the PrEP clinic; b) clinical eligibility form filled out during the clinical checkup that assessed the eligibility and intention to use PrEP; and c) the socio-behavioral questionnaire with information regarding lifestyles, sexual practices, experiences of discrimination and violence, and preventive methods for STIs, applied in the clinics by an interviewer, or self-administered by the participant if they choose to do so or because of the COVID-19 contingency plan [46].

### Study variables

The outcome variable was PrEP initiation defined by inclusion into the PrEP arm (PrEP prescription). The predictor variables for PrEP initiation were:

1. *Sociodemographic*: age (15 to 17 years; 18 and 19 years), a subpopulation (MSM; TGW), race/skin color (black—black and brown; non-black); schooling (at primary school; high school; higher education) and study site (Salvador, São Paulo, and Belo Horizonte); 2. *Sexual behavior*: unprotected sex at sexual debut (yes; no); unprotected anal sex in the last 6 months (yes; no); previous PEP use (yes; no);perceived risk of HIV infection on a scale of 0 to 10 (low—0 to 2; moderate—3 to 5; high—6 to 10); transactional sex in the previous three months (yes; no); use of hook-up apps in the previous three months (yes;no); an STI episode in the last 12 months (yes; no); frequent use of alcohol and/or drugs before or during sexual intercourse (yes; no); number of male casual partners in the previous three months (0 or 1; 2 to 5; 6 or more);3. *Violence and discrimination*: frequent experience of violence and discrimination due to sexual orientation or gender identity (yes; no); experience of sexual violence in a lifetime (yes; no).

### Data analysis

A descriptive analysis of the study population was conducted, as well as a bivariate analysis of sociodemographic and behavioral variables with PrEP initiation, stratified by the two age brackets. The variables with a p-value ≤0.05 or defined as relevant by their magnitude in the bivariate analysis were included in the final models. In the evaluation of the predictors of PrEP initiation, we fitted independent models yielding prevalence ratios (PR) and respective 95% confidence intervals (CI) using logistic regression models and the delta method for CI estimation [47]. Multicollinearity was analyzed using association tests between selected covariates for the models, and the adequacy of the final models was analyzed using the Hosmer-Lemeshow goodness-of-fit test [48], considering a cutoff p value of 0.05.

### Ethical consideration

The PrEP1519 study was conducted in accordance with the Brazilian (Resolution CNS no. 466, Brazil, 2012) and international research ethics guidelines, and it was approved by the ethics research committees (ERC) of the World Health Organization, Federal University of Bahia (#3,224,384), University of São Paulo (#3,082,360), and Federal University of Minas Gerais (#2,027,889). Written informed consent (WIC) was signed by 18 and 19-years old adolescents. For those aged <18 years, each city followed a different protocol according to local court decisions: for Belo Horizonte the WIC was signed by the parents or guardian as mandatory, followed by the assent form (AF) signed by the adolescents; for Salvador, there were two possibilities: i) the WIC was signed by a parent or guardian and the AF by the adolescent, or ii) the adolescent signed only the AF in cases in which the team’s psychologist and social worker judged that the family ties of the individual were broken or that the individual was at risk of physical, psychological, or moral violence due to the individual’s sexual orientation; and for São Paulo, only the AF was signed by the adolescents. All participants could withdraw consent at any stage of the process or skip any questions perceived as too sensitive, personal, or distressing. The data were stored in a secured database, and no personally identifiable information was used for any public presentation or publication to guarantee confidentiality.

## Results

Nine hundred-eight adolescents were linked to PrEP care and enrolled in the study. Among all adolescents linked to the PrEP clinic, 174 (19.2%) were between 15 and 17 years old, and 734 (80.8%) were between 18 and 19 years old. The majority self-identified as MSM (86.8% and 93.6%, respectively), as black (70.1% and 67.8%, respectively) and attended high school (74.7% and 66.2%, respectively). Other reported sexual behavior variables: frequent use of alcohol and/or drugs before or during sexual intercourse (31.5% and 32.5%, respectively); frequent experience of violence and discrimination due to sexual orientation or gender identity (33.3% and 33.0%, respectively); experience of sexual violence in a lifetime (30.0% and 27.2%, respectively); 2 to 5 sex partners in the previous three months (31.0% and 34.6%, respectively); unprotected sex at sexual debut (57.8% and 53.7%, respectively); unprotected anal sex in the previous six months (81.0% and 78.6%, respectively); use of hook-up apps in the last three months (63.8% and 71.0%, respectively); at least one STI episode in the last 12 months (16.1% and 22.6%, respectively); previously use of PEP (6.4% and 13.8%, respectively); 46.5% moderate risk perception of HIV infection and 20% high risk perception in both age groups; transactional sex in the previous three months (16.5% and 12.4%, respectively) (Table 1).

**Table 1:** Characteristics of the adolescent MSM and TGW enrolled in the PrEP1519 cohort by age groups. February 2019—February 2021.

Variable	Total (N = 908)		15 to 17 yo (N = 174)		18 and 19 yo (N = 734)	
	N	%	N	%	N	%
***Sociodemographic***						
**Subpopulation**						
MSM	838	92.3	151	86.8	687	93.6
TGW	70	7.7	23	13.2	47	6.4
**Race/ skin color**						
Non-black	288	31.7	52	29.9	236	32.2
Black	620	68.3	122	70.1	498	67.8
**Schooling**						
Primary school	60	6.6	37	21.3	23	3.1
High school	616	67.8	130	74.7	486	66.2
Higher education	232	25.5	7	4	225	30.7
**Study site**						
Salvador	301	33.2	47	27.0	254	34.6
São Paulo	416	45.8	76	43.7	340	46.3
Belo Horizonte	191	21.0	51	29.3	140	19.1
***Sexual behavior***						
**Unprotected sex at sexual debut**						
Yes	491	54.5	100	57.8	391	53.7
No	410	45.5	73	42.2	337	46.3
**Unprotected anal sex in the previous six months**						
Yes	718	79.1	141	81.0	577	78.6
No	190	20.9	33	19.0	157	21.4
**Previous PEP use**						
Yes	112	12.4	11	6.4	101	13.8
No	792	87.6	161	93.6	631	86.2
**Perceived risk of HIV infection**						
Low	295	32.9	57	33.5	238	32.7
Moderate	417	46.5	79	46.5	338	46.5
High	185	20.6	34	20.0	151	20.8
**Transactional sex in the previous three months**						
Yes	119	13.2	28	16.5	91	12.4
No	782	86.8	142	83.5	640	87.6
**Use of hook-up apps in the previous three months**						
Yes	632	69.6	111	63.8	521	71
No	276	30.4	63	36.2	213	29
**STI episode in the last 12 months**						
Yes	186	21.4	27	16.1	159	22.6
No	684	78.6	141	83.9	543	77.4
**Frequent use of alcohol and/or drugs before or during sexual intercourse**						
Yes	281	32.3	53	31.5	228	32.5
No	589	67.7	115	68.5	474	67.5
**Number of male casual partners in the previous three months**						
0–1	439	48.4	92	52.9	347	47.3
2–5	308	33.9	54	31.0	254	34.6
6 or more	161	17.7	28	16.1	133	18.1
***Violence and discrimination***						
**Frequent experience of violence and discrimination due to sexual orientation or gender identity**						
Yes	288	33.1	56	38.8	232	33
No	582	66.9	112	66.7	470	67
**Experience of sexual violence in a lifetime**						
Yes	248	27.7	51	30.0	197	27.2
No	647	72.3	119	70.0	528	72.8

Among adolescents linked to the PrEP clinics, 87.9% (798) indicated an intention to use oral PrEP, and 87.3% (793) were considered eligible to use PrEP at the first clinical visit. 704 (77.5%) initiated PrEP on the same day. And among the 798 who indicated an intention to use oral PrEP, 721 (90,4%) were considered eligible to start PrEP on the same day.

**Fig 1:**
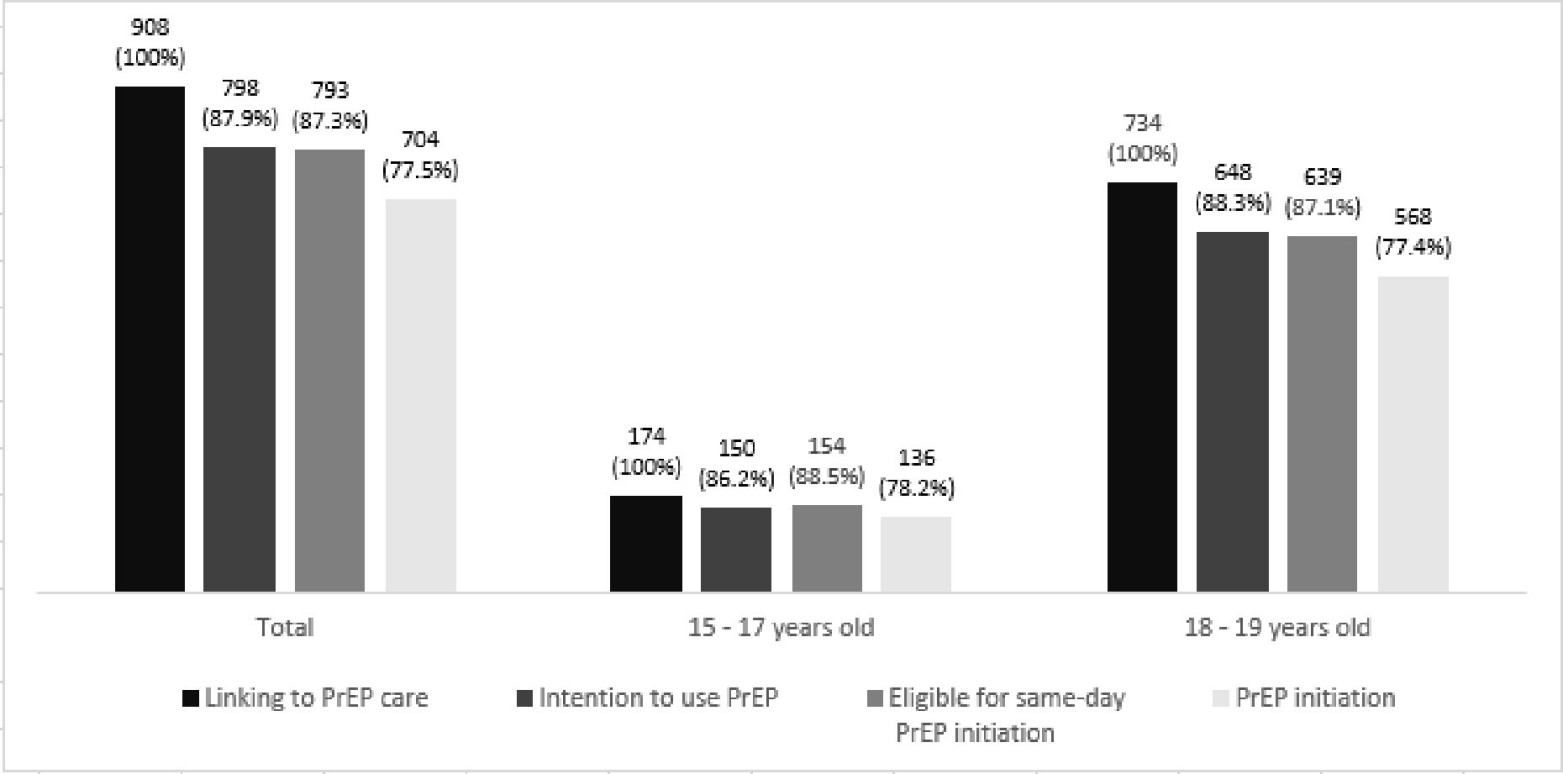
Intention to use, eligibility and PrEP initiation among adolescent MSM and TGW in the PrEP1519 cohort. February 2019—February 2021.

Among those 15 to 17 years old, 150 (86.2%) indicated an intention to use PrEP, 154 (88.5%) met the clinical criteria for PrEP use and were eligible for same-day PrEP initiation, and 136 (78.2%) initiated at the first visit. Among those 18 and 19 years old, 648 (88.3%) indicated an intention to use PrEP, 639 (87.1%) met the clinical criteria for PrEP use and were eligible for same-day PrEP initiation, 568 (77.4%) initiated at the first visit (Fig 1).

In the bivariate analysis, among adolescents aged 15 to 17, black adolescents (82.8%) and those who reported unprotected anal sex (83.7%) had a higher proportion of PrEP initiation (*p*<0.05). While among adolescents aged 18 and 19, a higher proportion of PrEP initiation (*p* < 0.05) occurred among those who had experienced violence and discrimination frequently due to sexual orientation or gender identity (83.2%); those who experienced sexual violence in a lifetime (83.2%); those who engaged in transactional sex in the previous three months (86.8%); those who had between 2 to 5 sexual partners in the previous three months (84.6%), and those who reported unprotected anal sex in the previous six months (81.5%) (Table 2).

**Table 2:** Bivariate analysis of PrEP initiation among adolescent MSM and TGW enrolled in the PrEP1519 cohort by age group. February 2019—February 2021.

*Variables*	*PrEP initiation*					
	15 to 17 yo			18 and 19 yo		
	N	%	p-value	N	%	p-value
	136/174	78.2	-	568/734	77.4	-
***Sociodemographic***						
** Subpopulation**			1.00			0.21
MSM	118	78.1		528	76.9	
TGW	18	78.3		40	85.1	
** Race/ skin color**			0.03			0.92
Non-black	35	67.3		182	77.1	
Black	101	82.8		386	77.5	
** Schooling**			0.10			0.45
Primary school	24	64.9		20	87.0	
High school	106	81.5		378	77.8	
Higher education	6	85.7		170	75.6	
** Study site**			0,02			0,06
Salvador	30	63,8		188	74.0	
São Paulo	62	81,6		262	77,1	
Belo Horizonte	44	86,3		118	84,3	
***Sexual behavior***						
** Unprotected sex at sexual debut**			0.35			0.66
Yes	81	81.0		305	78.0	
No	54	74.0		258	76.6	
** Unprotected anal sex in the previous six months**			0.001			<0.000
Yes	118	83.7		470	81.5	
No	18	54.5		98	62.4	
** Previous PEP use**			1,00			0.37
Yes	9	81.8		82	81.2	
No	126	78.3		484	76.7	
** Perceived risk of HIV infection**			0.23			0.17
Low	45	78.9		178	74.8	
Moderate	58	73.4		260	76.9	
High	30	88.2		125	82.8	
** Transactional sex in the previous three months**			0.80			0.02
Yes	23	82.1		79	86.8	
No	110	77.5		486	75.9	
** Use of hook-up apps in the previous three months**			0.13			0.06
Yes	91	82.0		413	79.3	
No	45	71.4		155	72.8	
** STI episode in the last 12 months**			1,00			0.75
Yes	21	77.8		124	78.0	
No	111	78.7		415	76.4	
** Frequent use of alcohol and/or drugs before or during sexual intercourse**			1,00			0.21
Yes	42	79,2		182	79.8	
No	90	78.3		357	75.3	
** Number of male casual partners in the previous three months**			0.52			0.001
0–1	69	75,0		251	72.3	
2–5	45	83,3		215	84,6	
6 or more	22	78.6		102	76.7	
***Violence and discrimination***						
** Frequent experience of violence and discrimination due to sexual orientation or gender identity**			0.84			0.00
Yes	45	80.4		193	83.2	
No	87	77.7		346	73.6	
** Experience of sexual violence in a lifetime**			0.69			0.03
Yes	41	80.4		164	83.2	
No	91	76.5		398	75.4	

In the multivariate analysis, a statistically significant association with PrEP initiation was found for black adolescents aged 15 to 17 years as compared to non-blacks (PR = 2.31; 95% CI = 1.10–4.84). And among adolescents aged 18 and 19, a statistically significant association with PrEP initiation was estimated for those who engaged in sex in exchange for money or favors in the last 3 months (PR 1.32 =; 95% CI = 1.04–1.68); those who experienced violence and discrimination due to sexual orientation or gender identity (PR = 1.21; 95% CI = 1.01–1.46); those who reported 2 to 5 male sexual partners during the last 3 months (PR = 1.39; 95% CI = 1.15–1.68). Unprotected anal sex in the last 6 months was associated with PrEP initiation in both age groups (15 to 17 years old: PR = 1.98; 95% CI = 1.02–3.85; 18 and 19 years old: PR = 1.45; 95% CI = 1.19–1.76) (Table 3).

**Table 3:** Multivariate analysis of PrEP initiation among adolescent MSM and TGW enrolled in the PrEP1519 cohort. February 2019—February 2021.

Variable		*PrEP initiation*		
	**15 to 17 yo**			**18 and 19 yo**
	**PR** *	**95% CI**	**PR** *	**95% CI**
**Race/ skin color**				
Non-black	1			
Black	2.31	[1.10–4.84]		
**Unprotected anal sex in the previous six months**				
No	1		1	
Yes	1.98	[1.02–3.85]	1.45	[1.19–1.76]
**Study site**				
Salvador	1		1	
São Paulo	2.31	[1.06–5.05]	0.99	[0.81–1.21]
Belo Horizonte	2.30	[1.05–5.04]	1.25	[0.99–1.58]
**Transactional sex in the previous three months**				
No			1	
Yes			1.32	[1.04–1.68]
**Frequent experience of violence and discrimination due to sexual orientation or gender identity**				
No			1	
Yes			1.21	[1.01–1.46]
**Experience of sexual violence in a lifetime**				
Yes			1	
No			1.12	[0.91–1.38]
**Use of hook-up apps in the previous three months**				
Yes			1	
No			0.91	[0.69–1.19]
**Number of male casual partners in the previous three months**				
0–1			1	
2–5			1.39	[1.15–1.68]
6 or more			1.01	[0.75–1.35]

* Regression Model adjusted by schooling.

## Discussion

The proportion of PrEP initiation was high among aMSM and aTGW who arrived at services and were enrolled in the cohort, demonstrating the interest and use among participants through active demand creation. This finding is consistent with other studies with adult MSM and TGW, in which PrEP initiation was greater than 60% [49–51]. There is a need to reach and inform a large number of adolescents from the key population about HIV prevention. Twenty percent of adolescent MSM and TGW reached out by the demand creation strategies of PrEP1519 did reach the PrEP clinics [45, 52]. Online interventions can be more cost-effective and easily scaled up to increase service coverage.

Face-to-face recruitment can reach proportionally more underserved adolescents from key populations. In combination, online and peer-driven face-to-face strategies can provide a critical balance between offering comprehensive coverage and equitable sexual health services for adolescents of key populations from different socioeconomic backgrounds [45].

The possibility of starting PrEP on the same day of the first visit may have contributed to the high rate of PrEP initiation among this population, as starting on the same day can increase uptake by reducing the time for PrEP initiation among individuals that are eligible and willing to use it [37, 38]. Studies show that the referral of adolescents to obtain a PrEP prescription at another moment, either in future clinical visits or at another site, causes losses in PrEP initiation rates [53, 54]. It is also important to emphasize that in our study, PrEP was widely promoted through demand creation strategies with the active role of peer educators (adolescents and young MSM or TGW). And the participants were cared for by a multidisciplinary team, which may have increased their PrEP initiation.

The number of black adolescents who started PrEP in the study was more significant than the number of non-blacks. In addition, among those aged 15 to 17 years, there was a strong association between PrEP initiation and self-reported black skin color, which may be related to the range of racial diversity recruitment strategies implemented in the study [45]; an important finding, as black MSMs and TGWs have been more exposed to HIV in several countries around the world [5, 55, 56] and in Brazil [57] due to social inequalities, racial discrimination, and less access to formal education, information, and health services [58, 59].Despite this, the rate of PrEP use in Brazil has been higher among white adults than among blacks [60].

Among adolescents aged 18 to 19, risky sexual practices (having unprotected sex, receiving money or favors in exchange for sex, and the number of sexual partners) and discrimination experiences were associated with PrEP initiation. In both age groups, having unprotected anal intercourse was associated with PrEP initiation, as seen in other studies with adult MSMs and TGWs [50, 61–65].

The practice of unprotected anal sex among adult MSMs and TGWs in Brazil is documented in the literature [17, 66, 67], despite the National Ministry of Health having several campaigns recommending the use of condoms for more than 3 decades [68]. The Brazilian Survey on Knowledge, Attitudes, and Practices among the Brazilian Population (PCAP), carried out in 2013, revealed that about a third of the adolescents and youth aged15 to 24 (36.9%) used a condom during sexual intercourse in the last 12 months, with steady and casual partners, and 19.5% had more than five casual partners in the last twelve months [69]. In our study, we observed a high proportion of unprotected anal sex among aMSM and aTGW, which may also reflect the tendency of a higher HIV risk among younger individuals. Thus, PrEP represents an important prevention strategy for this population.

Transactional sex can be an opportunity for MSM and TGW to have access to material goods and/or represent a means for survival [70, 71]. In this study, we chose to ask youth about receiving money or favors in exchange for sex as a way to indirectly identify those who had transactional sex. Notably, among people under 18, transactional sex represents sexual exploitation [72], defined as a heinous crime in Brazil [73], highlighting an essential problem with violations of fundamental rights among adolescents. In PrEP1519, the health team provided psychosocial care as well as the necessary referrals for minors that reported sexual exploitation. This situation may pose an increased risk of HIV infection [74, 75], as in some cases, the ability to decide on the use of condoms is limited or even discouraged, in response to offers of increased payment for the service [76, 77].

In our study, discrimination was identified as a factor associated with PrEP initiation among adolescents aged 18 to 19. The experience of discrimination due to gender identity and/or sexual orientation is still quite present among adolescents MSM and TGW, in our study as well as in a survey carried out with 521 MSM in the USA, in which about half of the participants revealed such experiences during adolescence [78]. Daily experiences with this discrimination and violence can negatively affect the health of aMSM and aTGW, affecting family bonds [79], the use of condoms in sexual relations (caused by the limited ability to negotiate their use with partners) [17, 80], job opportunities and access to goods and services, especially health care [81, 82].

Our study showed that to promote PrEP uptake and embrace marginalized adolescents who exchange sex for money or favors, who experience discrimination, violence and high-risk sexual practices, culturally sensitive health services that are attentive to the specific demands of the LGBTQIA+ population is fundamental [6, 81, 83, 84]. Therefore, it is increasingly necessary that health professionals are qualified, do not reproduce discriminatory practices at the service facilities [85], and offer a respectful environment, similar to the PrEP1519 care clinics.

During the period of this analysis, the COVID-19 pandemic impacted access to HIV prevention services among key populations in various countries [86, 87], increasing their vulnerability to HIV infection [88]. In the PrEP1519 study, we developed a contingency plan for maintaining the PrEP clinic open, which included the intensification of online recruitment strategies and telehealth during the pandemic [46]. Therefore, the quarantine and physical isolation measures did not significantly impact on the enrollment of adolescents. The proportion of enrolled participants was similar before and after the COVID-19 pandemic [45], as we quickly adapt the online strategies for the pandemic using social media and telemonitoring infrastructure.

Given the above, the PrEP uptake is high among aMSM and aTGW once the method is offered. Considering that these populations are among the most vulnerable to HIV in Brazil and the world [12], and their access to HIV prevention methods are still limited [16, 89], it is necessary to expand access to PrEP worldwide through strategies capable of identifying and linking adolescents in these contexts of vulnerability to offer PrEP.

### Limitations

Although we recognize the existence of essential differences between MSM and TGW, our study only included a small proportion of adolescent TGW due to the difficulty in accessing this population that is usually undergoing gender transition processes in this age group [90]. A similarly, it was observed during the enrollment of adolescents aged 15 to 17 years, given the legal requirements established in Salvador and Belo Horizonte cities to include minors in the study, mainly related to the need for consent from parents or legal guardians. The reimbursement for transportation and food costs is an incentive to reach and to link vulnerable adolescents MSM/TGW at high risk of HIV to the PrEP clinics, which may differ in PrEP services from the Brazilian National Health System. In addition, sexual practices, and experiences that are subject to social desirability bias were investigated. The interviewers were trained to maintain an objective and judgment-free language during the application of the questionnaires. Social desirability may have influenced the report of “intention to use PrEP”. Some adolescents may have expressed an intention to use PrEP because they had come to the PrEP clinic, received an incentive, and interacted with clinic staff, but did not intend to initiate PrEP on the same day. All interviews were conducted carefully, in a friendly space, and by an LGBTQIA+ friendly interviewer. The study excluded adolescents with mental illness, although they are also at HIV risk and may use PrEP. So, it is necessary also to investigate their PrEP initiation and use in the future.

## Conclusions

This study is the first to assess the PrEP uptake process and factors associated with PrEP initiation among aMSM and aTGW in Brazil. It was possible to observe that socio-behavioral conditions that represent a greater vulnerability to HIV and experiences of violence and discrimination were associated with PrEP initiation. These aspects highlight the need to inform and promote PrEP use, and other HIV combination prevention strategies among aMSM and aTGW at high HIV risk.

In addition, the biggest challenge to promoting the use of PrEP for aMSM and aTGW is in the first steps of the PrEP uptake process, as well as in accessing and linking to care. Once individuals are linked to the PrEP clinic, the prophylaxis initiation rates among them tend to be high. In this sense, the results indicate the challenges the services that offer this prevention strategy need to face as not only identifying but also reaching adolescents at higher HIV risk and promoting access to the prophylaxis, as well as motivating the continued use of PrEP, for whom this prevention strategy may be relevant at a given time in their lives.

Services can use the lessons learned from PrEP1519 to enable PrEP care access and link to HIV prevention, to facilitate effective demand creation strategies among these adolescents. A culturally sensitive approach, attentive to the specific demands of the LGBTQIA+ population is needed. In addition, it is necessary to organize the health service for the other stages of PrEP uptake, and promote the same-day PrEP initiation.
